# *N*-(1,5-Dimethyl-3-oxo-2-phenyl-2,3-dihydro-1*H*-pyrazol-4-yl)formamide

**DOI:** 10.1107/S1600536811015558

**Published:** 2011-05-07

**Authors:** Hao-Wei Wang, Ming-Ming Yang, Qi-Sheng Lu, Fang-Shi Li

**Affiliations:** a Department of Applied Chemistry, College of Science, Nanjing University of Technology, No. 5 Xinmofan Road, Nanjing 210009, People’s Republic of China

## Abstract

In the title compound, C_12_H_13_N_3_O_2_, the dihedral angle between the pyrazole and benzene rings is 50.0 (3)°. In the crystal, mol­ecules are linked by inter­molecular N—H⋯O hydrogen bonds to form a three-dimensional network. Two weak C—H⋯π inter­actions reinforce the crystal packing.

## Related literature

For bond-length data, see: Allen *et al.* (1987 ▶). For the preparation, see: Hosseini-Sarvari & Sharghi (2006 ▶).
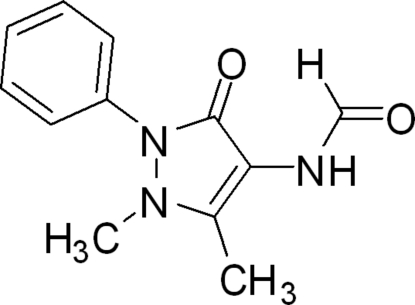


## Experimental

### 

#### Crystal data

C_12_H_13_N_3_O_2_
*M* *_r_* = 231.25
Orthorhombic, 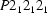

*a* = 8.4220 (17) Å
*b* = 9.2950 (19) Å
*c* = 14.501 (3) Å
*V* = 1135.2 (4) Å^3^
*Z* = 4
Mo *K*α radiation
μ = 0.10 mm^−1^
*T* = 293 K
0.20 × 0.10 × 0.10 mm

#### Data collection

Enraf–Nonius CAD-4 diffractometer
Absorption correction: ψ scan (North *et al.*, 1968 ▶) *T*_min_ = 0.981, *T*_max_ = 0.991
2320 measured reflections
2048 independent reflections
1327 reflections with *I* > 2σ(*I*)
*R*_int_ = 0.088
3 standard reflections every 200 reflections  intensity decay: 1%

#### Refinement

*R*[*F* ^2^ > 2σ(*F* ^2^)] = 0.064
*wR*(*F* ^2^) = 0.158
*S* = 1.01
2048 reflections
154 parameters
H-atom parameters constrained
Δρ_max_ = 0.19 e Å^−3^
Δρ_min_ = −0.25 e Å^−3^

### 

Data collection: *CAD-4 Software* (Enraf–Nonius, 1985 ▶); cell refinement: *CAD-4 Software*; data reduction: *XCAD4* (Harms & Wocadlo, 1995 ▶); program(s) used to solve structure: *SHELXS97* (Sheldrick, 2008 ▶); program(s) used to refine structure: *SHELXL97* (Sheldrick, 2008 ▶); molecular graphics: *SHELXTL* (Sheldrick, 2008 ▶); software used to prepare material for publication: *SHELXTL*.

## Supplementary Material

Crystal structure: contains datablocks I, global. DOI: 10.1107/S1600536811015558/bq2288sup1.cif

Structure factors: contains datablocks I. DOI: 10.1107/S1600536811015558/bq2288Isup2.hkl

Supplementary material file. DOI: 10.1107/S1600536811015558/bq2288Isup3.cml

Additional supplementary materials:  crystallographic information; 3D view; checkCIF report

## Figures and Tables

**Table 1:** Hydrogen-bond geometry (Å, °) *Cg*1 is the centroid of the C1–C6 ring.

*D*—H⋯*A*	*D*—H	H⋯*A*	*D*⋯*A*	*D*—H⋯*A*
N3—H3*A*⋯O1^i^	0.86	2.01	2.864 (5)	172
C10—H10*B*⋯*Cg*1^ii^	0.96	2.85	3.733 (5)	153
C12—H12*A*⋯*Cg*1^iii^	0.93	3.03	3.647 (5)	125

Symmetry codes: (i) 
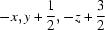
; (ii) 
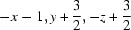
; (iii) 
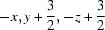
.

**Fig. 1.**
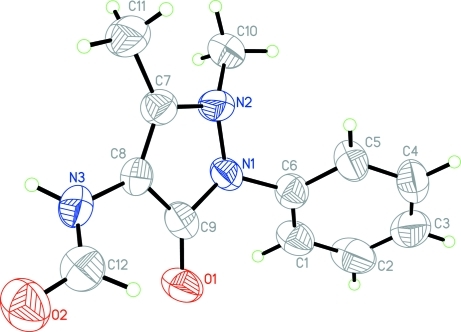
The molecular structure of (I), with the atom-numbering scheme. Displacement ellipsoids are drawn at the 50% probability level.

**Fig. 2.**
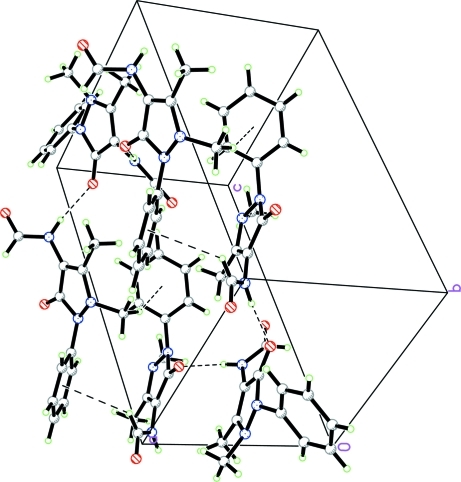
A packing diagram of (I). Hydrogen bonds are shown as dashed lines.

## supplementary crystallographic information

### Comment

The title compound, *N*-(1,5-dimethyl-3-oxo-2-phenyl-2,3-dihydro-1*H*-pyrazol-4-yl)formamide is an important intermediate for the synthesis of many drugs with antipyretic and analgesic effects. We report here the crystal structure of the title compound, (I).

The molecular structure of (I) is shown in Fig. 1. In the crystal, molecules are linked *via* intermolecular N—H···O hydrogen bond (Table 1) to form a three-dimensional network. The bond lengths and angles are within normal ranges (Allen *et al.*, 1987). The dihedral angle between the rings C1—C6) and (N1/N2/C7-C9) is 50.0 (3)°.

In the crystal, there are one intermolecular N—H···O hydrogen bond and two C—H···π interactions, one is between the methyl hydrogen and the phenyl ring, and the other is between the aldehyde hydrogen and the phenyl ring. The molecules are linked to each other by the intermolecular hydrogen bonds to form a three-dimensional network, which seem to be very effective in the stabilization of the crystal structure (Fig. 2.).

### Experimental

The title compound, (I) was prepared by the reaction of aminoantipyrin and formic acid in the presence of zinc oxide reported in literature (Hosseini-Sarvari & Sharghi, 2006). The crystals were obtained by dissolving (I) (0.2 g) in acetone (25 ml) and evaporating the solvent slowly at room temperature for about 5 d.

### Refinement

H atoms were positioned geometrically and refined as riding groups, with N—H = 0.86Å (for NH), C—H = 0.93, 0.93 and 0.96Å for aromatic, aldehydic and methyl H, respectively, and constrained to ride on their parent atoms, with *U*_iso_(H) = *xU*_eq_(C), where *x* = 1.2 for aromatic H, and *x* = 1.5 for other H.

### Crystal data

**Table:**

C_12_H_13_N_3_O_2_	*F*(000) = 488
*M**_r_* = 231.25	*D*_x_ = 1.353 Mg m^−^^3^
Orthorhombic, *P*2_1_2_1_2_1_	Mo *K*α radiation, λ = 0.71073 Å
Hall symbol: P 2ac 2ab	Cell parameters from 25 reflections
*a* = 8.4220 (17) Å	θ = 9–13°
*b* = 9.2950 (19) Å	µ = 0.10 mm^−^^1^
*c* = 14.501 (3) Å	*T* = 293 K
*V* = 1135.2 (4) Å^3^	Block, colorless
*Z* = 4	0.20 × 0.10 × 0.10 mm

### Data collection

**Table:**

Enraf–Nonius CAD-4 diffractometer	1327 reflections with *I* > 2σ(*I*)
Radiation source: fine-focus sealed tube	*R*_int_ = 0.088
graphite	θ_max_ = 25.3°, θ_min_ = 2.6°
ω/2θ scans	*h* = 0→10
Absorption correction: ψ scan (North *et al.*, 1968)	*k* = 0→11
*T*_min_ = 0.981, *T*_max_ = 0.991	*l* = −17→17
2320 measured reflections	3 standard reflections every 200 reflections
2048 independent reflections	intensity decay: 1%

### Refinement

**Table:**

Refinement on *F*^2^	Primary atom site location: structure-invariant direct methods
Least-squares matrix: full	Secondary atom site location: difference Fourier map
*R*[*F*^2^ > 2σ(*F*^2^)] = 0.064	Hydrogen site location: inferred from neighbouring sites
*wR*(*F*^2^) = 0.158	H-atom parameters constrained
*S* = 1.01	*w* = 1/[σ^2^(*F*_o_^2^) + (0.050*P*)^2^ + 0.950*P*] where *P* = (*F*_o_^2^ + 2*F*_c_^2^)/3
2048 reflections	(Δ/σ)_max_ < 0.001
154 parameters	Δρ_max_ = 0.19 e Å^−^^3^
0 restraints	Δρ_min_ = −0.25 e Å^−^^3^

### Special details

**Table:**

Geometry. All e.s.d.'s (except the e.s.d. in the dihedral angle between two l.s. planes) are estimated using the full covariance matrix. The cell e.s.d.'s are taken into account individually in the estimation of e.s.d.'s in distances, angles and torsion angles; correlations between e.s.d.'s in cell parameters are only used when they are defined by crystal symmetry. An approximate (isotropic) treatment of cell e.s.d.'s is used for estimating e.s.d.'s involving l.s. planes.
Refinement. Refinement of *F*^2^ against ALL reflections. The weighted *R*-factor *wR* and goodness of fit *S* are based on *F*^2^, conventional *R*-factors *R* are based on *F*, with *F* set to zero for negative *F*^2^. The threshold expression of *F*^2^ > σ(*F*^2^) is used only for calculating *R*-factors(gt) *etc*. and is not relevant to the choice of reflections for refinement. *R*-factors based on *F*^2^ are statistically about twice as large as those based on *F*, and *R*- factors based on ALL data will be even larger.

### Fractional atomic coordinates and isotropic or equivalent isotropic displacement parameters (Å^2^)

**Table:**

	*x*	*y*	*z*	*U*_iso_*/*U*_eq_	
O1	0.0661 (4)	0.6540 (3)	0.6577 (2)	0.0585 (9)	
N1	−0.0044 (4)	0.7935 (4)	0.5315 (2)	0.0464 (9)	
C1	−0.0412 (6)	0.5576 (5)	0.4655 (3)	0.0578 (12)	
H1A	−0.1048	0.5335	0.5157	0.069*	
O2	0.2871 (5)	0.8913 (4)	0.8637 (2)	0.0757 (11)	
N2	−0.0004 (5)	0.9417 (4)	0.5102 (2)	0.0524 (10)	
C2	−0.0105 (8)	0.4577 (5)	0.3988 (3)	0.0781 (18)	
H2A	−0.0536	0.3659	0.4031	0.094*	
N3	0.1224 (5)	0.9449 (4)	0.7471 (2)	0.0562 (10)	
H3A	0.0730	1.0081	0.7796	0.067*	
C3	0.0846 (8)	0.4939 (6)	0.3253 (4)	0.0777 (17)	
H3B	0.1059	0.4252	0.2803	0.093*	
C4	0.1490 (6)	0.6293 (6)	0.3167 (3)	0.0641 (14)	
H4A	0.2137	0.6524	0.2669	0.077*	
C5	0.1153 (6)	0.7298 (5)	0.3838 (3)	0.0593 (13)	
H5A	0.1556	0.8226	0.3787	0.071*	
C6	0.0215 (5)	0.6931 (5)	0.4588 (3)	0.0485 (11)	
C7	0.0387 (5)	1.0119 (4)	0.5892 (3)	0.0478 (11)	
C8	0.0694 (5)	0.9167 (4)	0.6560 (3)	0.0425 (10)	
C9	0.0471 (5)	0.7744 (5)	0.6213 (3)	0.0480 (11)	
C10	−0.1189 (6)	0.9958 (5)	0.4451 (3)	0.0610 (13)	
H10A	−0.1081	1.0982	0.4393	0.091*	
H10B	−0.2234	0.9731	0.4673	0.091*	
H10C	−0.1030	0.9515	0.3860	0.091*	
C11	0.0424 (7)	1.1725 (5)	0.5918 (3)	0.0712 (16)	
H11A	0.0750	1.2039	0.6519	0.107*	
H11B	−0.0615	1.2095	0.5785	0.107*	
H11C	0.1163	1.2073	0.5465	0.107*	
C12	0.2435 (6)	0.8781 (5)	0.7834 (3)	0.0592 (12)	
H12A	0.3008	0.8160	0.7457	0.071*	

### Atomic displacement parameters (Å^2^)

**Table:**

	*U*^11^	*U*^22^	*U*^33^	*U*^12^	*U*^13^	*U*^23^
O1	0.073 (2)	0.0519 (19)	0.0506 (18)	−0.0047 (16)	−0.0022 (16)	0.0143 (15)
N1	0.056 (2)	0.045 (2)	0.0383 (18)	−0.0037 (18)	0.0060 (16)	0.0059 (16)
C1	0.069 (3)	0.053 (3)	0.051 (3)	−0.012 (3)	−0.004 (2)	0.006 (2)
O2	0.087 (3)	0.080 (2)	0.060 (2)	−0.009 (2)	−0.0133 (19)	−0.0047 (19)
N2	0.071 (3)	0.0441 (19)	0.0423 (19)	0.011 (2)	−0.0070 (18)	0.0030 (17)
C2	0.127 (5)	0.051 (3)	0.057 (3)	−0.010 (3)	−0.021 (3)	0.000 (3)
N3	0.069 (3)	0.054 (2)	0.046 (2)	0.010 (2)	0.0007 (19)	−0.0117 (19)
C3	0.119 (5)	0.063 (3)	0.052 (3)	0.024 (4)	−0.016 (3)	−0.014 (3)
C4	0.060 (3)	0.087 (4)	0.045 (3)	0.006 (3)	0.007 (2)	−0.005 (3)
C5	0.067 (3)	0.064 (3)	0.047 (3)	−0.008 (3)	0.010 (2)	−0.001 (2)
C6	0.050 (3)	0.053 (3)	0.042 (2)	0.000 (2)	−0.004 (2)	−0.003 (2)
C7	0.050 (3)	0.046 (2)	0.047 (3)	0.003 (2)	−0.005 (2)	−0.001 (2)
C8	0.039 (2)	0.046 (2)	0.042 (2)	−0.0001 (19)	0.0031 (18)	−0.005 (2)
C9	0.044 (3)	0.061 (3)	0.039 (2)	−0.001 (2)	0.0013 (19)	0.004 (2)
C10	0.071 (3)	0.062 (3)	0.050 (3)	0.006 (3)	0.001 (2)	0.010 (2)
C11	0.101 (5)	0.048 (3)	0.065 (3)	0.000 (3)	−0.009 (3)	−0.007 (2)
C12	0.059 (3)	0.057 (3)	0.062 (3)	−0.004 (3)	−0.002 (3)	−0.003 (3)

### Geometric parameters (Å, °)

**Table:**

O1—C9	1.248 (5)	C3—H3B	0.9300
N1—C9	1.384 (5)	C4—C5	1.379 (6)
N1—N2	1.412 (5)	C4—H4A	0.9300
N1—C6	1.424 (5)	C5—C6	1.387 (6)
C1—C2	1.365 (6)	C5—H5A	0.9300
C1—C6	1.369 (6)	C7—C8	1.338 (5)
C1—H1A	0.9300	C7—C11	1.493 (6)
O2—C12	1.228 (5)	C8—C9	1.427 (6)
N2—C7	1.359 (5)	C10—H10A	0.9600
N2—C10	1.463 (5)	C10—H10B	0.9600
C2—C3	1.375 (8)	C10—H10C	0.9600
C2—H2A	0.9300	C11—H11A	0.9600
N3—C12	1.305 (6)	C11—H11B	0.9600
N3—C8	1.419 (5)	C11—H11C	0.9600
N3—H3A	0.8600	C12—H12A	0.9300
C3—C4	1.376 (7)		
			
C9—N1—N2	108.9 (3)	C5—C6—N1	120.4 (4)
C9—N1—C6	124.3 (3)	C8—C7—N2	109.8 (4)
N2—N1—C6	118.3 (3)	C8—C7—C11	129.7 (4)
C2—C1—C6	120.1 (5)	N2—C7—C11	120.4 (4)
C2—C1—H1A	119.9	C7—C8—N3	127.8 (4)
C6—C1—H1A	119.9	C7—C8—C9	109.4 (4)
C7—N2—N1	106.9 (3)	N3—C8—C9	122.7 (4)
C7—N2—C10	123.0 (4)	O1—C9—N1	123.6 (4)
N1—N2—C10	117.4 (4)	O1—C9—C8	131.7 (4)
C1—C2—C3	119.6 (5)	N1—C9—C8	104.7 (4)
C1—C2—H2A	120.2	N2—C10—H10A	109.5
C3—C2—H2A	120.2	N2—C10—H10B	109.5
C12—N3—C8	122.2 (4)	H10A—C10—H10B	109.5
C12—N3—H3A	118.9	N2—C10—H10C	109.5
C8—N3—H3A	118.9	H10A—C10—H10C	109.5
C2—C3—C4	121.6 (5)	H10B—C10—H10C	109.5
C2—C3—H3B	119.2	C7—C11—H11A	109.5
C4—C3—H3B	119.2	C7—C11—H11B	109.5
C3—C4—C5	118.3 (5)	H11A—C11—H11B	109.5
C3—C4—H4A	120.8	C7—C11—H11C	109.5
C5—C4—H4A	120.8	H11A—C11—H11C	109.5
C4—C5—C6	120.3 (5)	H11B—C11—H11C	109.5
C4—C5—H5A	119.8	O2—C12—N3	124.7 (5)
C6—C5—H5A	119.8	O2—C12—H12A	117.7
C1—C6—C5	120.1 (4)	N3—C12—H12A	117.7
C1—C6—N1	119.4 (4)		
			
C9—N1—N2—C7	−5.6 (5)	N1—N2—C7—C11	−175.9 (4)
C6—N1—N2—C7	−155.0 (4)	C10—N2—C7—C11	−35.6 (7)
C9—N1—N2—C10	−148.5 (4)	N2—C7—C8—N3	176.4 (4)
C6—N1—N2—C10	62.1 (5)	C11—C7—C8—N3	−3.6 (8)
C6—C1—C2—C3	0.5 (8)	N2—C7—C8—C9	−1.2 (5)
C1—C2—C3—C4	−0.5 (9)	C11—C7—C8—C9	178.8 (5)
C2—C3—C4—C5	−0.4 (8)	C12—N3—C8—C7	−130.2 (5)
C3—C4—C5—C6	1.4 (7)	C12—N3—C8—C9	47.1 (6)
C2—C1—C6—C5	0.5 (7)	N2—N1—C9—O1	−175.3 (4)
C2—C1—C6—N1	−176.6 (4)	C6—N1—C9—O1	−28.2 (6)
C4—C5—C6—C1	−1.5 (7)	N2—N1—C9—C8	4.8 (4)
C4—C5—C6—N1	175.6 (4)	C6—N1—C9—C8	151.9 (4)
C9—N1—C6—C1	61.7 (6)	C7—C8—C9—O1	177.7 (5)
N2—N1—C6—C1	−154.0 (4)	N3—C8—C9—O1	0.0 (7)
C9—N1—C6—C5	−115.4 (5)	C7—C8—C9—N1	−2.3 (5)
N2—N1—C6—C5	28.9 (6)	N3—C8—C9—N1	180.0 (4)
N1—N2—C7—C8	4.1 (5)	C8—N3—C12—O2	−174.8 (4)
C10—N2—C7—C8	144.5 (4)		

### Hydrogen-bond geometry (Å, °)

**Table:**

Cg1 is the centroid of the C1–C6 ring.

**Table:**

*D*—H···*A*	*D*—H	H···*A*	*D*···*A*	*D*—H···*A*
N3—H3A···O1^i^	0.86	2.01	2.864 (5)	172
C10—H10B···Cg1^ii^	0.96	2.85	3.733 (5)	153
C12—H12A···Cg1^iii^	0.93	3.03	3.647 (5)	125

Symmetry codes: (i) −*x*, *y*+1/2, −*z*+3/2; (ii) −*x*−1, *y*+3/2, −*z*+3/2; (iii) −*x*, *y*+3/2, −*z*+3/2.
